# Combined use of CEMIP and CA 19-9 enhances diagnostic accuracy for pancreatic cancer

**DOI:** 10.1038/s41598-018-21823-x

**Published:** 2018-02-21

**Authors:** Hee Seung Lee, Chan Young Jang, Sun A Kim, Soo Been Park, Dawoon E. Jung, Bo Ok Kim, Ha Yan Kim, Moon Jae Chung, Jeong Youp Park, Seungmin Bang, Seung Woo Park, Si Young Song

**Affiliations:** 1 Division of Gastroenterology, Department of Internal Medicine, Yonsei University College of Medicine, Seoul, Korea; 2 Institute of Gastroenterology, Yonsei University College of Medicine, Seoul, Korea; 3 Biostatistics Collaboration Unit, Yonsei University College of Medicine, Seoul, Korea

## Abstract

Carbohydrate antigen (CA) 19-9 is the only diagnostic marker used in pancreatic cancer despite its limitations. Here, we aimed to identify the diagnostic role of CEMIP (also called KIAA1199) combined with CA 19-9 in patients with pancreatic cancer. A retrospective analysis of prospectively collected patient samples was performed to determine the benefit of diagnostic markers in the diagnosis of pancreatic cancer. We investigated CEMIP and CA 19-9 levels in 324 patients with pancreatic cancer and 49 normal controls using serum enzyme-linked immunosorbent assay. Median CA 19-9 and CEMIP levels were 410.5 U/ml (40.8–3342.5) and 0.67 ng/ml (0.40–1.08), respectively, in patients with pancreatic cancer. The AUROC for CA 19-9 and CEMIP were 0.847 (95% confidence interval [CI]: 0.806–0.888) and 0.760 (95% CI: 0.689–0.831), respectively. Combination of CA 19-9 with CEMIP showed markedly improved AUROC over CA 19-9 alone in pancreatic cancer diagnosis (0.94 vs. 0.89; *P* < 0.0001). CEMIP showed a diagnostic yield of 86.1% (68/79) in CA 19-9 negative pancreatic cancer. Combined use with CEMIP showed significantly improved diagnostic value compared with CA 19-9 alone in pancreatic cancer. Especially, CEMIP may be a complementary marker in pancreatic cancer patients with normal CA 19-9 levels.

## Introduction

Pancreatic cancer is a highly malignant tumor with a poor prognosis. Most patients (80%) present with inoperable advanced pancreatic cancer at the time of initial diagnosis^1^. Although carbohydrate antigen (CA) 19-9 is known as a pancreatic cancer biomarker, it is not commonly used for general screening, owing to its low sensitivity and specificity^2–4^. In particular, false-negative results in the segment of the population with Lewis blood type A-B- and false-positive results in patients with obstructive jaundice limit the specificity of CA 19-9 for pancreatic cancer^5,6^. Therefore, development of novel diagnostic markers is required for the early detection of pancreatic cancer. Previous studies have suggested alternative biomarkers to compensate for the limitations of CA 19-9 in patients with pancreatic cancer^7–9^.

*CEMIP* was originally identified as a hearing loss-related gene. Increased expression of cell migration-inducing hyaluronan binding protein (CEMIP), a newly identified protein involved in hyaluronan degradation^10^, has been reported in various cancers. CEMIP contributes to breast cancer cell migration with induction of epithelial-mesenchymal transition via calcium signaling^11,12^. Additionally, CEMIP expression is associated with gastric and colorectal tumorigenesis and it is a potential biomarker of gastric cancer^13–15^.

Recently, several studies have investigated the role of CEMIP in pancreatic cancer; CEMIP has been reported to be associated with early detection, cancer cell migration, invasion, and poor prognosis in previous studies^10,16,17^. Suh *et al*. suggested that CEMIP may be useful for detecting pancreatic cancer at an early stage^16^, while Koga *et al*. demonstrated its association with prognosis in pancreatic cancer^10^. However, the sample sizes of these previous studies were too small to draw a solid conclusion^10,16,17^. On the other hand, despite its limitations, CA 19-9 has been proposed as a diagnostic tool for the detection of pancreatic cancer. To date, there have been no studies investigating the diagnostic accuracy of serum CA 19-9 in combination with CEMIP for pancreatic cancer, as compared with serum CA19-9 alone.

Therefore, we aimed to investigate the differences in CEMIP expression in whole blood between patients with pancreatic cancer and healthy participants, and to identify the role of CEMIP compared with that of CA 19-9 in the diagnosis of pancreatic cancer.

## Methods

### Patients

We performed a retrospective analysis of prospectively collected patient samples including pancreatic cancer patients and normal individuals between 2007 and 2015. As a control group, normal individuals were defined as people who were not diagnosed with pancreatic cancer, and these individuals were divided into two groups: healthy individuals and patients with benign diseases. Blood samples were obtained immediately following diagnosis and prior to the administration of any oncological or surgical treatment. Information regarding patient demographics and clinical data was obtained from the electronic medical records, including age at diagnosis, sex, location of cancer, serum levels of CA 19-9, antitumor treatment, and tumor stage. Tumor stages were based on the staging classification of the 7th edition of the American Joint Committee on Cancer (AJCC)^18^. Written informed consent was obtained from all patients who approved the use of their blood, and this study was approved by the institutional review board of Yonsei University.

### CA 19-9 and CEMIP

All methods were performed in accordance with relevant guidelines and regulations. CA 19-9 was measured using chemiluminescence immunoassay on the VITROS 3600 Immunodiagnostic System (Ortho-Clinical Diagnostics Inc., Raritan, NJ, USA). The standard diagnostic cutoff value for CA 19-9 was 37 U/mL. The levels of CEMIP were measured using the enzyme-linked immunosorbent assay (ELISA) method. We detected secreted CEMIP protein in blood samples obtained from patients with pancreatic cancer and individuals without cancer. A 96-well CEMIP ELISA kit (SER965Hu) was purchased from USCN Life Science, Inc. (Wuhan, China) and the procedure was performed according to the manufacturer’s instructions. The detection range for the ELISA kit used was 0.156–10 ng/ml. The standard curve concentrations used for the ELISAs were 10, 5, 2.5, 1.25, 0.625, 0.312, and 0.156 ng/ml. The sensitivity of this assay, or lower limit of detection (LLD), was defined as the lowest protein concentration that could be differentiated from zero. It was determined by adding two standard deviations to the mean optical density value of 20 standard replicates and calculating the corresponding concentration. No significant cross-reactivity or interference between CEMIP and analogues was observed.

### Statistical analyses

All statistical analyses were performed using SPSS for Windows version 20.0 (SPSS, Inc., Chicago, IL, USA), SAS (version 9.4, SAS Inc., Cary, NC, USA), and R package, version 3.0.1. For comparison of serum values between normal individuals and patients with pancreatic cancer, Student’s t test was employed. The receiver operating characteristic (ROC) curve and the area under the ROC curve (AUROC) were calculated to identify the optimal cut-off CEMIP level associated with diagnosis. We used MedCalc version 11.1 for the ROC analysis (MedCalc Software, Mariakerke, Belgium). The bootstrapping method (1,000 replications) was applied for internal validation of the AUROC. We used Contal and O’Quigley’s method of categorizing patients into high or low risk groups for overall survival and chose a cut-off point of CEMIP according to the method^19^. Survival curves were estimated using the Kaplan-Meier method, and the differences among curves were assessed using log-rank tests. A *P*-value < 0.05 was considered to be statistically significant. Univariate and multivariate analysis was performed using Cox regression analysis for risk factors influencing survival in patients who were diagnosed with pancreatic cancer. GraphPad Prism 4 software (GraphPad Software, San Diego, CA, USA) was used for construction of graphs.

## Results

### Patient characteristics

Serum samples were obtained from 324 patients diagnosed with pancreatic cancer and 49 normal individuals at the Severance Hospital. Normal individuals were divided into two groups: 30 healthy individuals and 19 patients with benign diseases such as common bile duct stone (n = 2), gallbladder stone (n = 4), intrahepatic stone (n = 1), cholecystitis (n = 4), liver abscess (n = 1), acute hepatitis (n = 1), acute pancreatitis (n = 1), duodenal polyp (n = 1), gastric ulcer (n = 1), and benign pancreatic cyst (n = 3). Table 1 shows the baseline characteristic of patients in the present study. The median age of patients was 63 years (men, 64.5%). About 75% of patients received palliative chemotherapy, and 170 patients (52%) were diagnosed with stage IV pancreatic cancer. The median overall survival was 314 days (169–581). The median levels of CA 19-9 and CEMIP were 410.5 U/ml (40.8–3342.5) and 0.67 ng/ml (0.40–1.08), respectively, in patients with pancreatic cancer.

**Table 1:** Baseline characteristics.

Variables	Patients (n = 324)
Age	63.0 ± 10.6
Men	209 (64.5%)
Location	
Head	168 (51.9%)
Body	66 (20.4%)
Tail	58 (17.9%)
Mixed	32 (9.8%)
Antitumor treatment	
Operation	
Whipple procedure	4 (1.2%)
PPPD	47 (14.5%)
Total pancreatectomy	1 (0.3%)
Distal pancreatectomy	13 (4.0%)
Chemotherapy	245 (75.6%)
Supportive care	14 (4.3%)
Stage	
I	4 (1.2%)
II	89 (27.5%)
III	61 (18.8%)
IV	170 (52.5%)
CEMIP, ng/mL	0.67 (0.40–1.08)
CA 19–9, U/mL	410.5 (40.8–3342.5)
Overall survival	314 days (169–581)

Variables are expressed as mean ± standard deviation, median (interquartile range), or n (%). PPPD, pylorus-preserving pancreaticoduodenectomy; CEMIP, cell migration-inducing hyaluronan binding protein; CA 19-9, carbohydrate antigen 19-9.

### CEMIP as diagnostic marker

**Figure 1:**
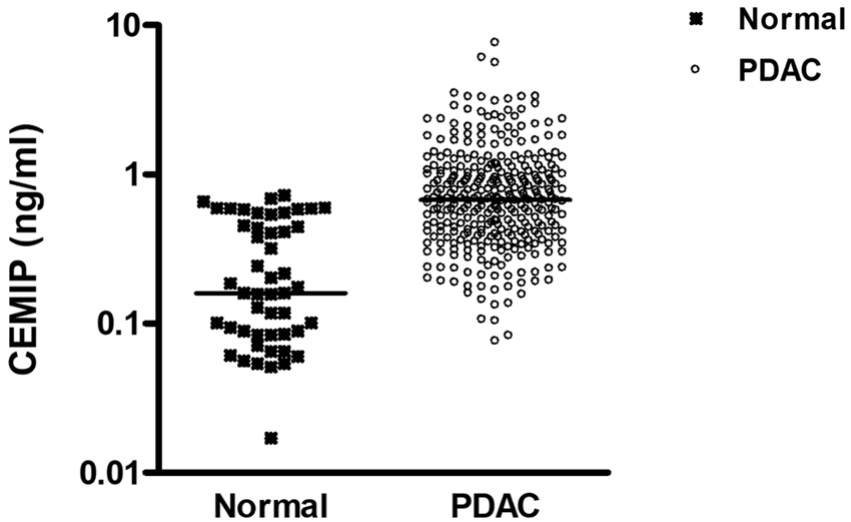
Summary of serum CEMIP expression levels detected from pancreatic cancer patients and individuals without cancer. The CEMIP expression was significantly higher in patients with pancreatic cancer than in individuals without cancer (P < 0.001 by paired t-test). The horizontal lines represent the median values. CEMIP, cell migration-inducing hyaluronan binding protein; PDAC, pancreatic ductal adenocarcinoma.

Using the Mann-Whitney U test, the CA 19-9 levels were found to be significantly elevated in the serum of patients with pancreatic cancer compared with normal individuals (median, 410.5 vs. 10.8 U/ml, *P* < 0.001). Moreover, CEMIP also exhibited significantly higher expression levels in patients with pancreatic cancer than in normal individuals (0.67 vs. 0.16 ng/ml, *P* < 0.001) (Fig. 1).

**Figure 2:**
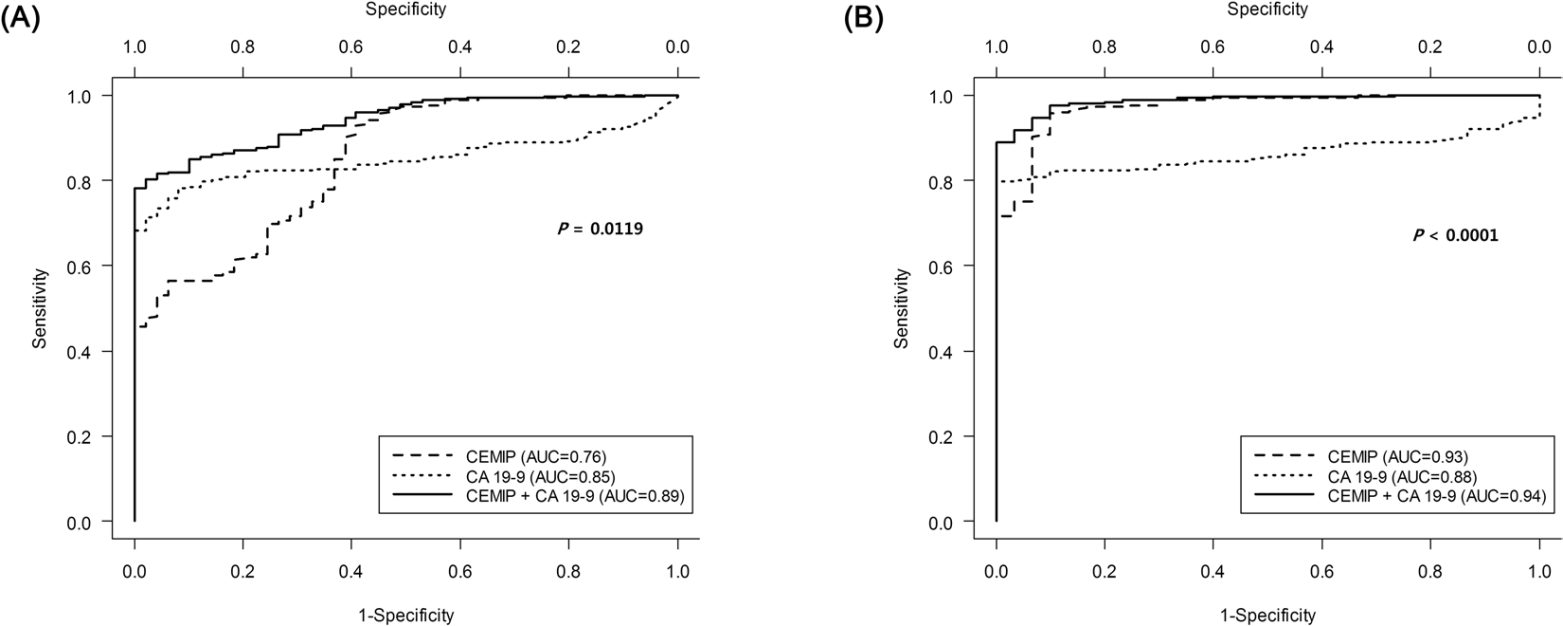
ROC curves of CEMIP, CA 19-9, and both to diagnose pancreatic cancer. Normal individuals were defined as people who were not diagnosed with pancreatic cancer and were divided into two groups (healthy individuals and patients with benign disease). (**A**) Normal individuals including healthy individuals and patients with benign disease were the control group. Combination with CEMIP showed markedly improved AUROC over that of CA 19-9 alone in the diagnosis of pancreatic cancer against normal individuals (AUROC, 0.89 vs. 0.85; *P* = 0.0119). (**B**) Healthy individuals were the control group. Combination with CEMIP showed markedly improved AUROC over that of CA 19-9 alone in the diagnosis of pancreatic cancer against healthy individuals (AUROC, 0.94 vs. 0.89; *P* < 0.0001). CEMIP, cell migration-inducing hyaluronan binding protein; CA 19-9, carbohydrate antigen 19-9; ROC, receiver operating characteristic; AUC, area under the ROC curve.

The ROC curve analysis indicated the potential diagnostic values of these markers (Fig. 2): The AUROCs for CA 19-9 and CEMIP were 0.85 (95% confidence interval [CI]: 0.806–0.888) and 0.76 (95% CI: 0.689–0.831), respectively. CEMIP also showed good diagnostic performance in internal validation (bootstrap-corrected AUROC, 0.76; 95% CI, 0.692–0.833). Combined ROC curve analysis using CA 19-9 and CEMIP revealed an AUROC of 0.89 (95% CI: 0.862–0.920) with a sensitivity of 80.3% and a specificity of 97.9% in discriminating pancreatic cancer patients from normal individuals. Combination of CA 19-9 with CEMIP showed markedly improved AUROC over that of CA 19-9 alone in the diagnosis of pancreatic cancer against normal individuals (AUROC, 0.89 vs. 0.85; *P* = 0.0119) (Fig. 2A). With combined CEMIP and CA 19-9, the test showed better sensitivity and negative predictive value compared to CA 19-9 alone (Table 2).

**Table 2:** Performance characteristics of CA 19-9 and CEMIP.

Test	Sensitivity (%)	Specificity (%)	PPV (%)	NPV (%)
CA 19-9	75.6	93.8	98.7	36.8
CEMIP	92.9	59.2	93.7	55.7
CEMIP + CA 19-9	96.6	59.2	94.0	72.5

PPV, Positive predictive value; NPV, Negative predictive value; CA 19-9, Carbohydrate antigen 19-9.

Additional subgroup analysis was performed by including only healthy individuals among all normal individuals. Combination of CA 19-9 with CEMIP showed markedly improved AUROC over that of CA 19-9 alone in the diagnosis of pancreatic cancer against healthy individuals (AUROC, 0.94 vs. 0.88; *P* < 0.0001) (Fig. 2B).

### CEMIP in CA 19-9 negative patients

**Figure 3:**
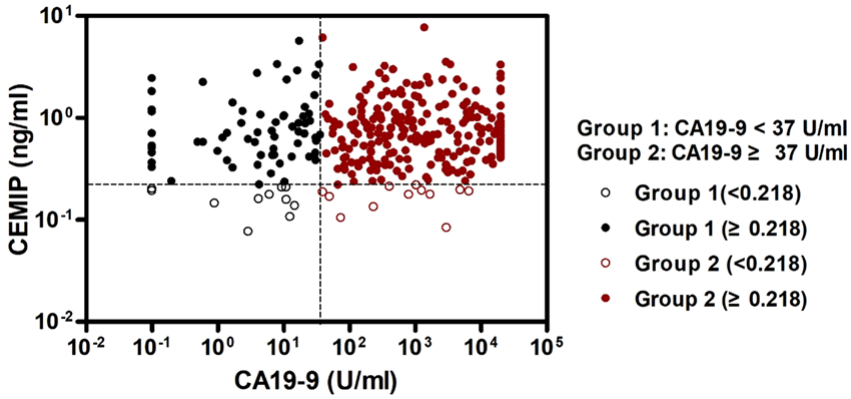
Distribution of CEMIP and CA 19-9 levels in patients diagnosed with pancreatic cancer. The optimal cut-off levels of CA 19-9 and CEMIP were 37 U/mL and 0.218 ng/ml, respectively. A total of 68 CA 19-9 negative patients (black colored circle) were diagnosed using CEMIP (diagnostic yield, 86.1%). CEMIP, cell migration-inducing hyaluronan binding protein; CA 19-9, carbohydrate antigen 19-9.

Among 324 patients who were diagnosed with pancreatic cancer, 79 patients demonstrated CA 19-9 levels below 37 U/ml, and 19 patients were Lewis blood type A-B-. The proportion of patients who showed high CEMIP levels (≥0.218 ng/ml) among pancreatic cancer patients with CA 19-9 in the normal range (<37 U/ml) was 86.1% (Supplementary Table 1). Among 79 patients with CA 19-9 in the normal range, 68 patients (black colored circle) were diagnosed using CEMIP (diagnostic yield, 86.1%) (Fig. 3).

### CEMIP as prognostic predictive marker

**Figure 4:**
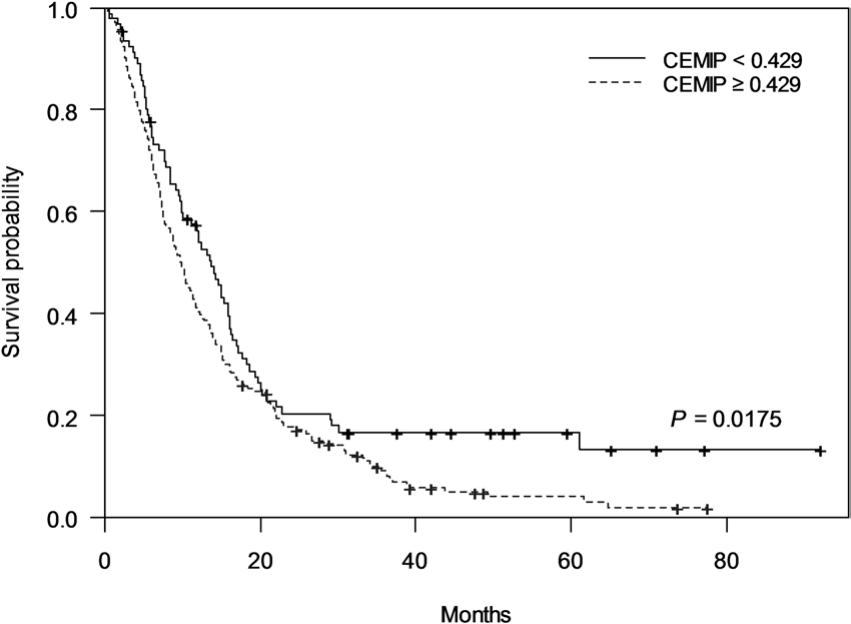
Correlation between CEMIP and survival in patients with pancreatic cancer using Kaplan-Meier curve. Low CEMIP levels were significantly associated with longer overall survival (median overall survival, 13.7 vs. 9.8 months, *P* = 0.0175). CEMIP, cell migration-inducing hyaluronan binding protein.

We selected the optimal cut-off value of CEMIP to maximize the survival difference using Contal and O’Quigley’s method (CEMIP = 0.429 ng/ml). Using Kaplan-Meier curves, we examined the correlation between CEMIP expression and survival in patients with pancreatic cancer. Survival analysis revealed a significantly shorter overall survival in the high CEMIP expression group (≥0.429 ng/ml) compared with the low CEMIP expression group (<0.429 ng/ml) (9.8 vs. 13.7 months, log-rank test *P* = 0.0175) (Fig. 4).

To further determine the prognostic value of CEMIP expression for pancreatic cancer, clinicopathological variables as well as CEMIP expression status were analyzed using Cox proportional hazard models. Univariate analysis revealed significant prognostic factors including high CEMIP expression, age, anticancer treatment, and tumor stage (*P* < 0.05). Multivariate analysis revealed that high CEMIP expression (hazard ratio [HR], 1.646; 95% CI, 1.224–2.214; *P* = 0.001) was a significant independent prognostic factor after adjustment for potential confounding factors (Table 3). In 2 patients whose CEMIP and CA 19-9 levels were serially checked two times with tumor response evaluation, CEMIP showed a predictive function for treatment response, and it accurately predicted cancer progression compared to CA 19-9 (Supplementary Figure 1).

**Table 3:** Univariate and multivariate analysis for risk factors influencing survival in patients diagnosed with pancreatic cancer.

Variable	Univariate		Variable	Multivariate	
	HR (95% CI)	*P*-value		HR (95% CI)	*P*-value
CEMIP, mg/dL			CEMIP, mg/dL		
<0.429	Ref.		<0.429	Ref.	
≥0.429	1.378 (1.056-1.798)	0.018	≥0.429	1.646 (1.224–2.214)	0.001
Age	1.022 (1.010–1.035)	0.001	Age	1.022 (1.009–1.036)	0.001
Sex					
Women	Ref.				
Men	1.219 (0.957–1.552)	0.108			
Diabetes mellitus					
No	Ref.				
Yes	0.947 (0.729–1.229)	0.680			
Treatment			Treatment		
Supportive care	Ref.		No	Ref.	
Chemotherapy	0.521 (0.280–0.970)	0.039	Yes	0.243 (0.128–0.462)	<0.001
Operation	0.134 (0.069–0.263)	<0.001			
Location of cancer					
Head	Ref.				
Body	1.372 (1.013–1.859)	0.041			
Tail	1.444 (1.049–1.986)	0.024			
Mixed	1.344 (0.883–2.045)	0.167			
Stage			Stage		
I and II	Ref.		I and II	Ref.	
III	1.228 (0.862–1.748)	0.254	III	1.733 (1.137–2.639)	0.011
IV	3.754 (2.807–5.020)	<0.001	IV	5.310 (3.663–7.696)	<0.001

A *P-*value < 0.02 was considered statistically significant. We found the cut-off point to maximize the survival curve using Contal and O’Quigley’s method.

HR, hazard ratio; CI, confidence interval.

## Discussion

This study describes a novel biomarker, CEMIP, which may be used in combination with CA 19-9 in whole blood in the diagnosis of pancreatic cancer. CEMIP synergizes with CA 19-9 in its diagnostic function for pancreatic cancer. Combining CA 19-9 with CEMIP increased the AUROC compared with CA 19-9 alone in all groups. These results suggest the usefulness of CEMIP in combination with CA 19-9 or in situations in which the CA19-9 level is normal.

Cancer stem cells (CSCs) are potential diagnostic and therapeutic targets because of their roles in carcinogenesis^20,21^. In recent research, CSC-related biomarkers have been suggested as potential reliable biomarkers for tumor detection^22,23^. We previously performed cDNA microarrays using adherent and sphere cells from the human pancreatic cancer cell lines Capan-1 and HPAC to evaluate the unique molecular patterns of pancreatic CSCs^24^. We characterized biomarkers for distinguishing pancreatic CSCs from cancer cells. cDNA microarrays with sphere and adherent cells derived from Capan-1 and HPAC cell lines revealed that the *CEMIP* gene was upregulated in sphere cells, but not in adherent Capan-1 and HPAC cells (data not shown, Jang *et al*., submitted). Among the candidate genes identified in this work, we were particularly interested in the *CEMIP* gene, which has not been studied extensively in CSCs^21,25,26^. We focused on the role of CEMIP in relation to CSC-related functions, especially diagnostic and prognostic function. CSC-related markers have been shown to aid early detection in cancer and have been associated with chemotherapy resistance and prognosis, because CSCs are known as tumor-initiating cells and conventional anticancer treatments do not target CSCs^20–23,27–29^.

Combining detection with different biomarkers has been reported to be a useful strategy in several studies, as it increases the sensitivity and specificity of each biomarker. In a combined ROC analysis using the two markers, both sensitivity and specificity reached a relatively high value in discriminating patients with pancreatic cancer from healthy individuals. Recent studies reported the effect of combined metabolic biomarkers or microRNA based molecular markers in pancreatic cancer diagnosis^8,9^. However, no reliable CSC-related biomarker has been established for pancreatic cancer diagnosis and prognosis combined with widely used CA 19-9. Our results suggest that the combination of CA 19-9 and CEMIP serum assays may offer a useful tool for the detection of pancreatic cancer.

Regarding early-stage pancreatic cancer screening, endoscopic ultrasound, magnetic resonance imaging, and computed tomography have been used in previous studies for several decades as a first choice in patients with high risk factors^30–37^. However, those procedures are associated with low compliance in the clinical setting due to their invasive and expensive character. Therefore, cost-effective and non-invasive biomarkers with high sensitivity and specificity are needed to enable early detection of early-stage pancreatic cancer. Thus, we evaluated the diagnostic performance of CEMIP in patients with stage I & II pancreatic cancer. In early-stage pancreatic cancer, combined use of CEMIP and CA 19-9 showed higher AUROC than CA 19-9 alone (0.95 vs. 0.85, *P* = 0.0004) (Supplementary Figure 2).

There are several strengths in the present study. First, this study is the largest to investigate the role of CEMIP in pancreatic cancer. Second, this study is based on the finding that CEMIP is associated with CSCs. No previous studies have investigated the association between CEMIP and CSCs or the role of CEMIP as a CSC-related marker. Third, all clinical data and blood samples were collected from a large-scale prospective cohort of patients with pancreatic cancer.

However, there are also several limitations in this study. First, owing to restrictions in the sampling of healthy participants, there was a significant difference in age (*P* < 0.001) between patients with pancreatic cancer and normal individuals (mean age, 63 vs. 49 years). Thus, the outcome might be confounded by age for healthy blood donors. However, separate analysis for cases and controls revealed no correlation between age and CEMIP (Supplementary Figure 3). This finding suggests that CEMIP might be a useful diagnostic biomarker independent of age. In addition, the number of individuals in the control groups is small compared to the number in the group with pancreatic cancer. Future prospective studies with large sample sizes including healthy individuals and patients with premalignant disease may further support our results. Lastly, the specificity of combination of CEMIP and CA19-9 was low (59.2%) even though the sensitivity was high (96.6%). Although the specificity of CEMIP was high (90.0%) to identify patients with pancreatic cancer when compared to the normal controls without benign diseases, it became low probably due to the high number of false-positive cases. Therefore, clinicians should be careful to use CEMIP and CA 19-9 as biomarkers for pancreatic cancer diagnosis in patients with benign disease. For these patients, other examinations including endoscopic ultrasonography and abdominal computed tomography scan might be helpful to complement the low specificity of the combination of CEMIP and CA 19-9.

In conclusion, serum ELISA analysis showed that CEMIP proteins were highly expressed in patients with pancreatic cancer compared to healthy individuals. Combined use of CA 19-9 and CEMIP significantly increased the sensitivity and specificity in discriminating not only patients with all stage pancreatic cancer but also patients with stage I/II pancreatic cancer from healthy individuals. Therefore, combined detection with serum CA 19-9 and CEMIP levels may have the potential to become a new laboratory method for the clinical diagnosis of pancreatic cancer.

## Electronic supplementary material

Supplementary Information
