# Supplementary material for: Combined use of CEMIP and CA 19-9 enhances diagnostic accuracy for pancreatic cancer
Source: Sci Rep. 2018 Feb 21;8:3383. doi: 10.1038/s41598-018-21823-x (PMC5821821; doi:10.1038/s41598-018-21823-x)

Combined use of CEMIP and CA 19-9 enhances diagnostic accuracy for pancreatic cancer

Short title: CEMIP as a novel tumor marker of pancreatic cancer

Hee Seung Lee[1], Chan Young Jang[1], Sun A Kim[1], Soo Been Park[1],

Dawoon E. Jung[2], Bo Ok Kim[3], Ha Yan Kim[3], Moon Jae Chung[1],

Jeong Youp Park[1], Seungmin Bang[1], Seung Woo Park[1], Si Young Song[1]

[1]*Division of Gastroenterology, Department of Internal Medicine,*

Yonsei University College of Medicine, Seoul, Korea

[2]*Institute of Gastroenterology, Yonsei University College of Medicine*

[3]*Biostatistics Collaboration Unit, Yonsei University College of Medicine, Seoul, Korea*

Corresponding author:

Si Young Song, MD, PhD

Division of Gastroenterology, Department of Internal Medicine,

Yonsei University College of Medicine,

50-1 Yonsei-ro, Seodaemun-gu, Seoul 03722, Korea

Phone: +82-2-2228-1957

Fax: +82-2-2227-7900

E-mail: sysong@yuhs.ac

Supplementary Table 1. Proportion of patients with high CEMIP levels in patients with pancreatic cancer with normal range CA 19-9 and Lewis A-/B-

	CA 19-9 < 37 U/mL	Lewis A-/B-
CEMIP > 0.218 ng/mL	86.1% (68/79)	89.5% (17/19)

* The results for Lewis A/B were available in 64 patients.

Supplementary Figure 1. Representative cases for CEMIP level and its predictive function for tumor response.

Patient (A)	Feb. 2012	Apr. 2012
Tumor marker		
CA 19-9, U/mL	6150	1980 ↓
CEMIP, ng/mL	1.119	2.061 ↑
CT findings		Increased malignant ascites in abdomen and slightly enlarged pancreatic mass

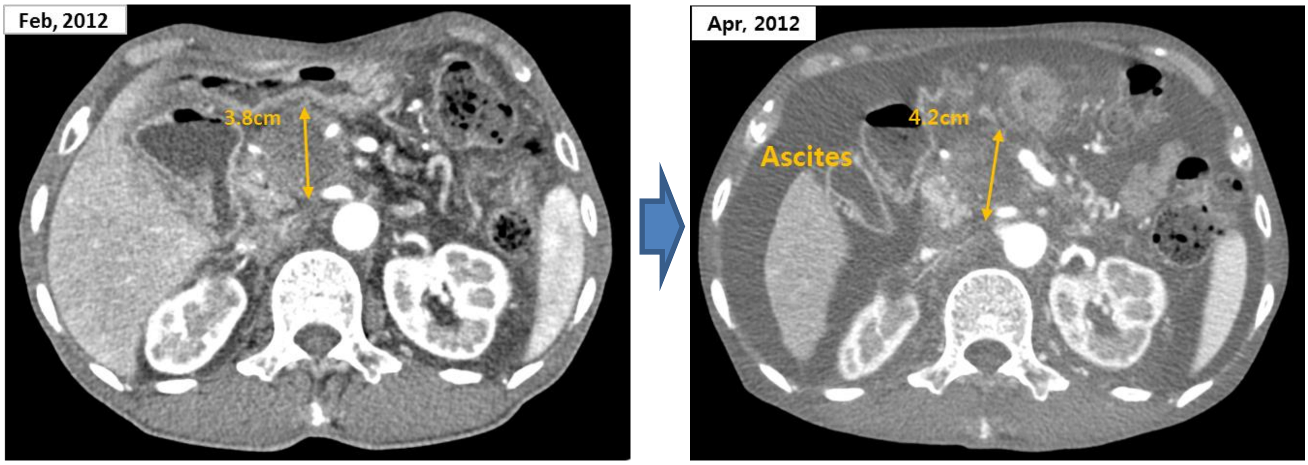

Patient (B)	Dec. 2008	Feb. 2009	May. 2009
Tumor marker			
CA 19-9, U/mL	100	85.5	63.5 ↓
CEMIP, ng/mL	0.487		1.639 ↑
CT findings			Newly developed metastatic mass in liver dome

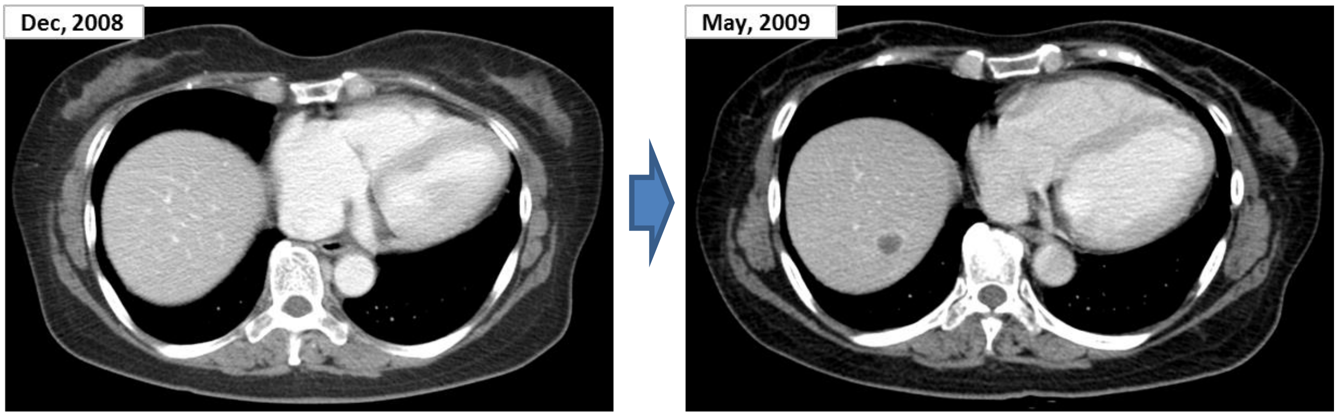

Supplementary Figure 2. ROC curves of CEMIP, CA 19-9, and both for diagnosis of early-stage pancreatic cancer (stage I & II). Combined use of CEMIP and CA 19-9 showed higher AUC than CA 19-9 alone (0.95 vs. 0.85, *P* = 0.0004).

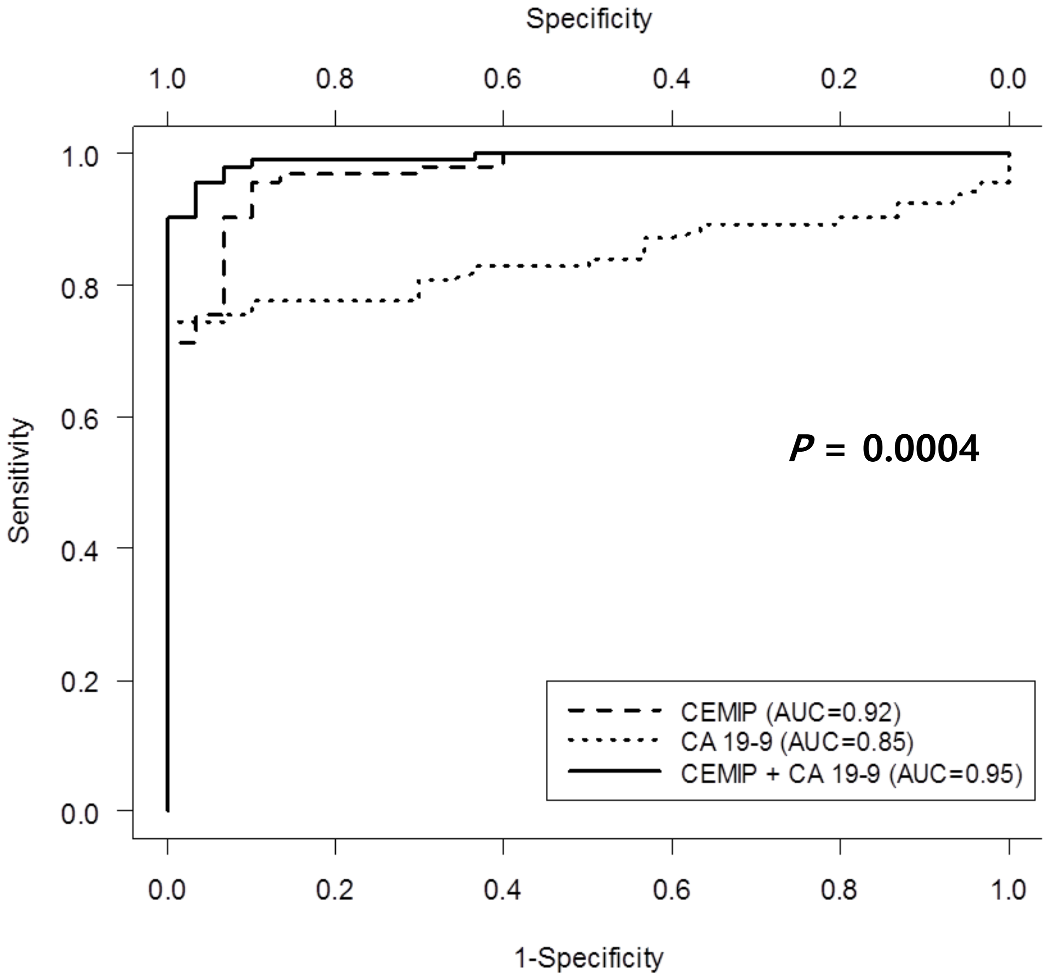

Supplementary Figure 3. (A) CEMIP level by age (< 65 and ≥ 65 years) in participants. Median score is the line in the middle of the box and the 25th and 75th percentile are the lower and upper part of the box. Outliers are given as circles. Pearson correlation coefficient = 0.1597, P = 0.002. (B) Scatter plot between CEMIP level and age.

(A)

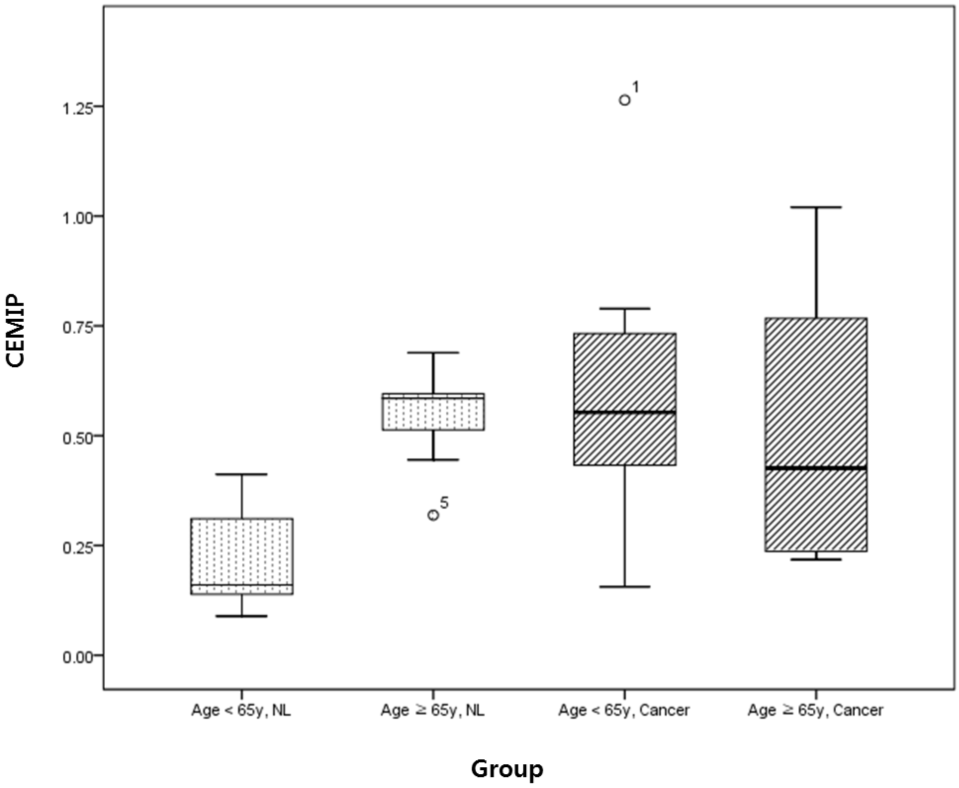

(B)

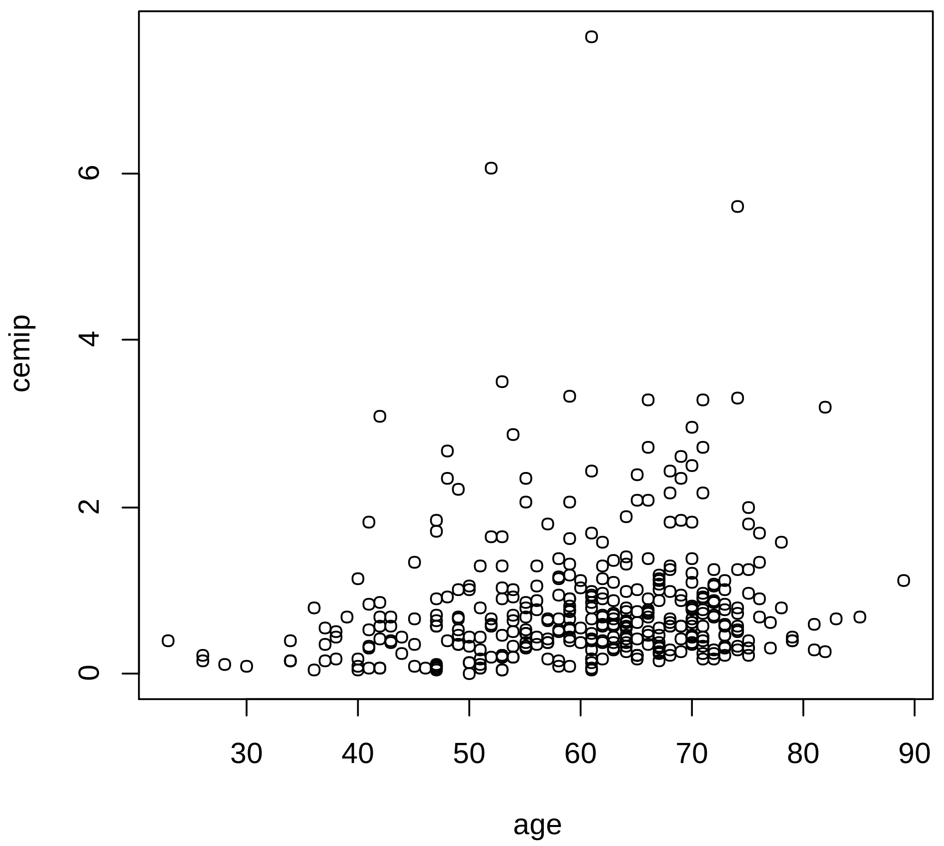

Supplement: Supplementary file 1 — Supplementary Information [file 41598_2018_21823_MOESM1_ESM.pdf]
